# Living with coeliac disease in unsupportive systems: a qualitative study of women’s free-text narratives

**DOI:** 10.1016/j.ijnsa.2026.100632

**Published:** 2026-07-14

**Authors:** Julián Rodríguez-Almagro, Cristina Romero-Blanco, Antonio Hernández-Martínez, María Laura Parra-Fernandez, Maria Dolores Onieva-Zafra, Alberto Bermejo-Cantarero

**Affiliations:** a PhD. Department of Nursing, Ciudad Real School of Nursing. University of Castilla La-Mancha, Ciudad Real, Spain; b Instituto de Investigación Sanitaria de Castilla-La Mancha (IDISCAM), Toledo, Spain

**Keywords:** Coeliac disease, Women’s experiences, Qualitative descriptive study, Everyday disease management, Psychosocial burden, Nursing care

## Abstract

**Background:**

Coeliac disease requires lifelong dietary management that extends beyond biomedical treatment and profoundly shapes everyday life across social, emotional, and structural domains.

**Aim:**

To explore how women living with coeliac disease describe the everyday experience of managing the condition using large-scale free-text narratives.

**Design:**

Qualitative descriptive study.

**Methods:**

Free-text responses were drawn from a large national survey conducted in Spain of 310 women aged 18–65 years with a medical diagnosis of coeliac disease confirmed by a gastroenterologist. Data were analyzed using reflexive thematic analysis, focusing on recurring patterns of meaning across narratives rather than individual trajectories, consistent with the nature of open-ended survey data.

**Results:**

Four interrelated themes were identified. Home was described as a space of safety, while public and social food environments were experienced as sites of vulnerability. Everyday management was characterized by constant vigilance and emotional exhaustion driven by fear of gluten contamination. Social restriction, stigma, and feeling like a burden shaped relationships and participation. Economic burden, limited institutional support, and difficulties navigating food and social environments perceived as insufficiently protective of women with coeliac disease further compounded daily management, with food frequently framed as a form of treatment lacking adequate systemic backing.

**Conclusion:**

Coeliac disease was experienced by women as sustained everyday work embedded in ongoing social negotiation and structural constraints, underscoring the potential need for more supportive social, food service, and healthcare systems. We have highlighted the need for nursing and health service responses that move beyond dietary instruction to address psychosocial burden, risk management in social eating contexts, and structural sources of inequity.

**What is already known about the topic**

• Coeliac disease requires lifelong adherence to a gluten-free diet and is associated with psychosocial burden beyond symptom control.
• Eating outside the home is a recurring source of perceived risk, vigilance, and social restriction for people living with coeliac disease.
• Qualitative research has largely relied on small samples, limiting insight into shared experiential patterns across diverse social contexts.

**What this paper adds:**

• Drawing on 310 free-text narratives from women, we identified four interrelated experiential mechanisms shaping everyday management of coeliac disease.
• We have reconceptualised the gluten-free diet as sustained psychosocial and organisational work rather than a purely nutritional treatment.
• Participants described coeliac disease management as a sustained form of emotional, social, and organisational work shaped by vigilance, stigma, and limited structural support.

## 1 Introduction

Coeliac disease is a chronic autoimmune disorder triggered by the ingestion of gluten in genetically predisposed individuals and requires lifelong adherence to a strict gluten-free diet (Aljada et al., 2021). While the gluten-free diet is effective in controlling the disease from a biomedical perspective, living with coeliac disease extends far beyond the avoidance of a single ingredient. It reshapes food-related practices, daily routines, social relationships, and the ways in which individuals navigate public and private spaces. In this sense, living with coeliac disease involves a continuous process of managing food-related risk and adapting to changing everyday contexts(White, Bannerman and Gillett, 2016)

Beyond its dietary and clinical dimensions, coeliac disease has been described as a form of biographical disruption, marking a clear “before and after” in individuals’ lives (Bury, 1982) . Qualitative researchers have indicated that receiving a diagnosis often initiates a complex process of identity reconfiguration, in which relief at finally naming longstanding symptoms coexists with fear, uncertainty, and the perceived loss of spontaneity in everyday life (Olsson et al., 2009; Rose, Law and Howard, 2024). Living with coeliac disease, therefore, entails an ongoing effort to re-establish a sense of normality within social and everyday environments that are frequently unprepared for the condition (Rodríguez-Almagro, 2016)

A growing body of qualitative research has shown that, even in contexts where the availability of gluten-free products has increased, everyday life with coeliac disease remains characterized by uncertainty, constant vigilance, and substantial cognitive burden related to eating safely. Qualitative researchers have described persistent patterns of risk anticipation, loss of spontaneity, changes in the relationship with food, and the progressive experience of the disease as a pervasive burden extending beyond the clinical domain (Rose, Law and Howard, 2024). In an integrative review of living with a gluten-free diet, Almagro et al. (2018) highlighted the role of social support, stigma, and institutional arrangements in shaping daily disease management. Economic factors—particularly the cost and accessibility of gluten-free foods— have been shown to further compound this burden and are frequently framed by patients as an inequitable aspect of treatment (Kowalczuk and Moor, 2025).

These ongoing precautionary measures are particularly intensified outside the home. Qualitative researchers examining adaptation to the gluten-free diet have shown that eating shifts from an automatic habit to a deliberate practice requiring planning, continuous label reading, and constant evaluation of potential sources of contamination, especially in social and public settings (Garnweidner-Holme et al., 2020; Dalton and Castellanos, 2023). As a result, everyday food practices become embedded in broader processes of anticipation and negotiation that extend well beyond individual dietary choices.

A central experiential feature emerging from this literature is food-related hypervigilance (Wolf et al., 2018). The need to continuously anticipate risk, manage cross-contamination, and negotiate food-related decisions transforms eating into a cognitively and emotionally demanding practice(Olsson et al., 2009; Rose, Law and Howard, 2024). Over time, this sustained state of alertness has been described as mentally exhausting and anxiety-provoking, shaping not only how individuals relate to food but also how they engage in social interactions and navigate public spaces (Hallert, Sandlund and Broqvist, 2003; Kowalczuk and Moor, 2025).

The social dimension of coeliac disease plays a central role in shaping these experiences. Eating in public settings can transform an otherwise invisible condition into a visible one, requiring explanations, special requests, and ongoing negotiation with the surrounding environment. Qualitative researchers have shown that these dynamics may generate discomfort, experiences of stigma, and relational burden, negatively affecting social participation and the sense of normality in everyday life (Olsson et al., 2009).

Researchers have also suggested possible sex-related differences in the psychosocial experience of coeliac disease. Women may report greater emotional burden, food-related vigilance, and stigma-related distress associated with dietary management and social eating contexts (Sverker et al., 2009; Rodriguez-Almagro, 2019). These differences may also relate to broader gendered expectations surrounding food preparation, caregiving, and responsibility for dietary management within everyday social contexts.These findings support the importance of further exploring women’s lived experiences of coeliac disease within everyday social and structural contexts.

Structural and contextual factors further intersect with individual experiences of coeliac disease. Researchers have suggested that increased commercial availability of gluten-free products does not necessarily reduce individual responsibility or the emotional burden associated with dietary risk management, which continues to fall largely on the person living with the condition (Jansson-Knodell et al., 2023) . Changes in policies related to access to gluten-free foods or financial support have been shown to intensify practical concerns and perceptions of inequity, reinforcing the understanding of dietary treatment as a sustained structural demand rather than a simple clinical recommendation (Crocker, Jenkinson and Peters, 2018).

Together, these experiential dimensions underscore why living with coeliac disease cannot be fully understood through biomedical indicators alone but requires attention to the lived, relational, and structural contexts in which dietary treatment is enacted. Despite this growing body of evidence, there remains a need for qualitative studies that explore, in a broad and naturalistic manner, how coeliac disease is experienced in everyday life, identifying shared patterns of meaning derived from individuals’ own narratives. In this context, open-ended responses embedded within surveys constitute a valuable source of qualitative data, as they allow participants to articulate concerns, experiences, and priorities spontaneously, without the constraints of an interview guide. When analyzed using reflexive thematic analysis, such data can yield robust interpretations of common experiential mechanisms across the dataset (Braun and Clarke, 2006, 2014; Kiger and Varpio, 2020).

While interview-based qualitative studies have provided rich, in-depth accounts of living with coeliac disease, less attention has been paid to the analytic potential of large-scale free-text narratives embedded within survey research. Recent methodological work supports the use of open-ended survey responses to identify shared experiential patterns across broad samples, particularly when the aim is to capture recurrent pressures and concerns rather than individual trajectories (Cunningham and Wells, 2017; Abraham et al., 2020; Neate et al., 2022; Norberg et al., 2023; Sezgin et al., 2023; Young, Young and Field, 2024; Steele et al., 2025).

Taken together, existing qualitative evidence highlights that living with coeliac disease involves ongoing cognitive, emotional, social, and structural work that extends far beyond dietary restriction alone. However, there remains a need for qualitative research that captures, in a broad and naturalistic manner, how these demands are articulated in individuals’ own words, identifying shared experiential patterns across large and diverse samples. Open-ended survey responses offer a valuable opportunity to explore everyday concerns and meanings as they are spontaneously expressed by people living with the condition.

The aim of the present study was to explore the lived experience of women with coeliac disease through qualitative analysis of open-ended survey responses, identifying recurring themes and shared patterns of meaning related to dietary management, emotional burden, social interaction, and structural constraints shaping everyday life with the condition.

## 2 Methods

Ethical approval was obtained from the Social Research Ethics Committee of XXXXX. All participants provided informed consent prior to participation, and qualitative data were analyzed in anonymized form in accordance with ethical and data protection requirements.

### 2.1 Study design

We used a qualitative descriptive design to provide a comprehensive, low-inference account of how women describe living with coeliac disease in their own terms (Sandelowski, 1995, 2000, 2010; Colorafi and Evans, 2016). Qualitative description is particularly appropriate in applied health research when the aim is to generate clinically and service-relevant insights that remain closely grounded in participants’ language, without imposing high levels of theoretical abstraction (Sandelowski, 2000; Colorafi and Evans, 2016) .

The study was based on patient-generated written narratives collected through an open-ended survey question. The use of free-text survey responses as qualitative material is increasingly common in health research and can yield rich experiential data when analyzed systematically (Hall, Rubin and Charnock, 2009; Neate et al., 2022; Norberg et al., 2023).

The open-ended question was designed to invite participants to freely describe experiences, concerns, and challenges related to everyday life with coeliac disease, consistent with the exploratory aims of the study. The survey instrument was reviewed by the research team prior to dissemination to ensure clarity and suitability of the wording for capturing experiential narratives.

### 2.2 Data source and participants

Qualitative data were obtained from an open-ended question included in a large national survey conducted in Spain involving women diagnosed with coeliac disease. All respondents were invited to provide written comments describing their experiences, concerns, or challenges related to daily life with coeliac disease.

Participants were recruited through multiple dissemination channels, including coeliac disease associations, social media platforms, and online patient networks. The survey was distributed nationally across Spain using convenience sampling methods aimed at reaching adults living with coeliac disease in community settings rather than through clinical services alone.

Eligible participants were adult women living in Spain who self-reported a medical diagnosis of coeliac disease confirmed by a gastroenterologist and who completed the open-ended survey question. No additional exclusion criteria were applied to the qualitative dataset. Information regarding comorbid conditions or additional dietary restrictions was not systematically collected for the purposes of the present analysis and, therefore, could not be examined in relation to participants’ narratives.

A total of 310 women provided at least one free-text response and were included in the qualitative analysis. Basic sociodemographic and clinical characteristics of participants included in the qualitative dataset are presented in Table 1 to contextualise the sample in relation to existing literature. The sample included women with varying times since diagnosis and duration of adherence to a gluten-free diet, supporting exploration of shared experiential patterns across different stages of living with coeliac disease. Responses varied in length and depth, ranging from brief statements to detailed narratives. No minimum length was required for inclusion, as all responses were considered meaningful expressions of participants’ perspectives (Abraham et al., 2020; Neate et al., 2022; Steele et al., 2025).

**Table 1:** Sociodemographic and clinical characteristics of participants included in the qualitative analysis (*N* = 310).

Variable	Qualitative sample (*N* = 310)
Age, years, mean (SD)	36.2 (11.1)
Educational level, *n* (%)	
• Low	9 (2.9%)
• Medium	65 (20.9%)
• High	236 (76.2%)
Body mass index, kg/m², mean (SD)	23.1 (4.3)
Time since diagnosis, years, median (IQR)	7.0 (3.0–15.0)
Dietary adherence score (3–15)*, mean (SD)	4.3 (1.6)
Employed, *n* (%)	221 (71.3%)
Married or living with partner, *n* (%)	198 (63.9%)
Member of a coeliac association, *n* (%)	143 (46.1%)
Self-reported financial difficulties related to gluten-free diet, *n* (%)	176 (56.8%)

**Abbreviations:** IQR = interquartile range; *N* = total sample size; *n* = number of participants; SD = standard deviation.

⁎ Lower scores indicate better dietary adherence.

We focused the present analysis exclusively on the qualitative narratives with the aim of identifying shared patterns of meaning rather than comparing subgroups. The qualitative dataset was overwhelmingly composed of female participants and, given previous literature suggesting possible sex-related differences in the psychosocial experience of coeliac disease, the analysis was restricted to women to preserve analytic coherence and support a more focused exploration of shared experiential patterns (Sverker et al., 2009; Rodriguez-Almagro, 2019)

Responses were treated as qualitative units of meaning rather than as individual interview cases. Demographic variables were not used to stratify the qualitative analysis, as the aim was to identify shared patterns of experience across narratives rather than to compare subgroups (Sandelowski, 2000; Colorafi and Evans, 2016) .

All free-text responses were exported from the survey dataset and anonymized prior to analysis. Responses were screened to remove any direct identifiers. The qualitative dataset was then compiled into an analysis file, preserving the original wording and punctuation to maintain closeness to participants’ language (Sandelowski, 2000; Colorafi and Evans, 2016) .

### 2.3 Data analysis

Qualitative data were analyzed using reflexive thematic analysis, following the phases outlined by Braun and Clarke (2006, 2014, 2019). Reflexive thematic analysis was chosen because it is well suited to identifying recurring patterns of meaning across large qualitative datasets and supports transparent, iterative theme development (Braun and Clarke, 2014, 2019). Its flexibility makes it particularly appropriate for analyzing large sets of written qualitative data generated through open-ended survey responses.

According to Braun and Clarke (2014, 2019), the analytic process involved the following:

1. Familiarization with the data through repeated reading of all free-text responses.
2. Inductive coding, focusing on meanings related to daily disease management, emotional impact, social interactions, and perceived support.
3. Generation of preliminary themes by identifying recurring patterns across responses.
4. Iterative refinement of themes to ensure internal coherence, distinctiveness, and explanatory relevance.
5. Selection of illustrative quotations that clearly represented each theme.

Coding was conducted inductively and iteratively through repeated engagement with the dataset. Initial coding was primarily undertaken by two members of the research team with experience in qualitative health research and coeliac disease-related research. Codes and preliminary themes were subsequently discussed among the wider research team to support reflexive interpretation and thematic refinement. Analysis was conducted manually using annotated text documents and coding matrices rather than qualitative data analysis software, consistent with the descriptive and interpretive aims of the study.

As the qualitative material consisted of open-ended survey responses rather than interviews, analysis prioritized the identification of shared patterns of meaning across narratives rather than the reconstruction of individual trajectories or longitudinal accounts (Hall, Rubin and Charnock, 2009; Neate et al., 2022; Norberg et al., 2023). Themes were developed inductively to capture how participants described the broader impact of coeliac disease on daily life, particularly in relation to responsibility for disease management, emotional burden, social participation and interactions with healthcare and institutional contexts.

Given the use of open-ended survey responses rather than iterative qualitative data collection, the concept of theoretical saturation was not applied in a traditional sense (Malterud, Siersma and Guassora, 2016; Norberg et al., 2023). Instead, analytic sufficiency was assessed through the recurrence, coherence, and explanatory relevance of patterns of meaning across the dataset, consistent with qualitative studies analyzing free-text survey data (Malterud, Siersma and Guassora, 2016; Dalal Albudaiwi, 2017). Themes were considered robust when they were consistently identified across multiple narratives and demonstrated relevance in relation to the study aims.

The analysis focused on identifying shared experiential patterns across narratives rather than comparing subgroups. While gender may shape aspects of lived experience in coeliac disease, the present study was not designed to examine gender-based contrasts, as the data were not generated through purposive sampling or analytic strategies aimed at subgroup comparison (Braun and Clarke, 2006, 2014; Campbell et al., 2021). The use of free-text survey responses as qualitative data is similarly oriented toward capturing experiential patterns across samples, rather than enabling robust subgroup comparisons (Neate et al., 2022; Norberg et al., 2023) .

Future qualitative research specifically designed to explore gendered experiences of coeliac disease, using purposive sampling and in-depth qualitative methods, would be warranted to examine potential differences in greater depth(Rodrigo-Sáez et al., 2011).

### 2.4 Reflexivity and analytical positioning

The analysis was conducted using a reflexive, interpretive stance consistent with reflexive thematic analysis, acknowledging that themes are generated through researchers’ active engagement with the data rather than discovered as objective entities (Braun and Clarke, 2019). Accordingly, analysis was understood as an interpretive process shaped by researchers’ perspectives, disciplinary backgrounds and analytic decisions. The goal was not theory generation but the production of a coherent, clinically and practice-relevant thematic account grounded in participants’ descriptions (Sandelowski, 2000; Colorafi and Evans, 2016)

The research team included investigators with backgrounds in nursing, chronic illness research, quality-of-life research, and qualitative health methodologies. Several authors had prior experience conducting research on coeliac disease and psychosocial aspects of chronic conditions, which informed sensitivity to issues related to stigma, vigilance, and everyday disease management during interpretation of the narratives. Reflexivity was maintained through ongoing analytic discussions within the research team, during which interpretations and thematic constructions were critically reviewed in relation to researchers’ assumptions, disciplinary perspectives, and familiarity with the topic area.

Members of the coeliac community did not directly participate in the analytic process or review of the final manuscript. Although the study was grounded in patient-generated narratives, the absence of direct patient involvement in interpretation and manuscript review may be considered a limitation and should be taken into account when interpreting the findings.

### 2.5 Trustworthiness

We aimed to enhance quality and transparency through explicit reporting of analytic procedures and systematic linking of interpretations to the dataset using verbatim quotations (Korstjens and Moser, 2018; Johnson, Adkins and Chauvin, 2020). Credibility was supported by analyzing the full set of available free-text responses, attending to convergence and recurrence across the dataset, and ensuring that themes were grounded in multiple excerpts rather than isolated statements (Korstjens and Moser, 2018; Johnson, Adkins and Chauvin, 2020).

### 2.6 Ethical considerations

The study was conducted in accordance with the Declaration of Helsinki (World Medical Association, 2024). Ethical approval was obtained from the relevant institutional review board. All participants provided informed consent prior to participation, and qualitative data were analyzed in anonymized form to protect confidentiality. All free-text responses were anonymized prior to analysis through removal of direct identifying information. The dataset was stored on password-protected institutional computers accessible only to the research team. In accordance with institutional data protection procedures and applicable data protection regulations, anonymized research data will be securely retained for the period required by institutional policy and subsequently destroyed. This study was approved by the Social Research Ethics Committee of the University of Castilla-La Mancha CEIS-2024–36,901).

## 3 Results

The reflexive thematic analysis of 310 free-text responses identified four interconnected themes describing how women with coeliac disease experience everyday life beyond adherence to a gluten-free diet. Together, these themes illustrated a sustained state of vigilance, embodied uncertainty, social and identity-related costs, and the perceived lack of structural support, revealing how disease management permeates physical, emotional, and social domains (Table 2).

**Table 2:** Qualitative themes and subthemes derived from free-text survey responses.

Theme	Subtheme	Analytical description
Theme 1. Living in a state of perpetual alert: everyday life as a space of risk	1.1. Home as the only space of control	Home is described as the only environment where food-related risks can be fully controlled. External environments require trust in others and are perceived as psychologically unsafe.
	1.2. Constant anticipation and loss of spontaneity	Everyday activities involving food require planning, anticipation, and risk assessment, leading to a persistent state of alertness and reduced spontaneity.
Theme 2. The body as a site of uncertainty and fear	2.1. Fear of bodily reactions	Even with strict dietary adherence, the body is perceived as unpredictable, generating ongoing fear of physical reactions and loss of bodily trust.
	2.2. Emotional exhaustion from bodily vigilance	Continuous monitoring of bodily sensations and potential symptoms leads to cumulative emotional fatigue over time.
Theme 3. Social and identity cost of living with coeliac disease	3.1. Social restriction and withdrawal	Difficulties managing food in social settings lead to avoidance of social activities and progressive withdrawal from shared eating experiences.
	3.2. Minimisation by others and narrative exhaustion	Repeated minimisation of the disease by others generates fatigue from constant explanation and defence of legitimacy, affecting identity and social belonging.
Theme 4. Living without a safety net: economic burden and lack of structural support	4.1. Gluten-free diet as costly treatment	Gluten-free food is framed as a medical necessity rather than a lifestyle choice, creating significant financial burden.
	4.2. Perceived lack of institutional support	Participants report limited economic, professional, and institutional support, reinforcing a sense of managing the disease alone.

Note. Quotes are derived from open-ended responses included in the survey and were selected to illustrate recurring patterns of meaning identified through reflexive thematic analysis.

Theme 1 Living in a state of perpetual alert: everyday life as a space of risk.

Participants frequently described daily life as dominated by constant anticipation and monitoring, particularly in relation to food. The home environment emerged as the only space of reliable control, where risks could be actively managed and uncertainty reduced. As one participant noted: *“In my own kitchen I feel safe”.* By contrast, eating outside the home was consistently associated with doubt and psychological strain: *“At home everything is controlled, but eating out always brings psychological consequences: ‘Will this be contaminated [with gluten]?’”* and *“Outside, you just have to trust, and that’s very hard.”*

This heightened vigilance extended beyond practical food choices and shaped women’s experience of time, agency, and spontaneity. Expressions such as *“You are always alert”* and *“always thinking about contamination”* were pervasive, indicating that everyday social participation is continuously constrained by the need to anticipate risk.

Theme 2 The body as a site of unpredictability and fear.

Beyond environmental vigilance, narratives revealed a persistent sense of embodied uncertainty. Even with strict adherence to a gluten-free diet, many women reported a lingering fear of physical reactions, encapsulated in statements like *“It’s not just the diet; it’s the fear of getting sick that never really goes away.”* This fear functioned as an internal monitoring system, directing attention toward bodily sensations and potential signs of exposure, regardless of actual gluten intake.

Expressions such as *“Will this be contaminated?”* and *“always thinking about what you can eat and what you can’t”* underscored how the body became a source of vigilance and anxiety in its own right, rather than a passive site of symptoms. This interplay between diet, embodied risk, and emotional burden contributed to a sustained psychological load, persisting even for participants long accustomed to managing the disease.

Theme 3 The social and identity cost of living with coeliac disease.

The social implications of the disease emerged as a central dimension of lived experience. Many women described a progressive narrowing of their social worlds, often as a self-protective strategy to avoid discomfort, conflict, or misunderstanding. One participant explained: *“I’ve stopped going out with friends because they don’t want to look for restaurants where I can eat.”* This social contraction reflected both practical barriers and emotional fatigue associated with negotiating dietary needs in group contexts.

Another recurring pattern involved minimization by others, wherein participants were told things like *“For one day, nothing will happen”* or accused of exaggerating their condition. Such interactions provoked a sense of wearing down — a narrative exhaustion stemming from the relentless need to justify and defend one’s illness: *“They tell you that you are exaggerating.”* The cumulative effect shaped participants’ sense of identity as misunderstood, burdensome, or othered, reinforcing social withdrawal and emotional strain.

Theme 4 Living without a safety net: economic burden and lack of structural support.

A prominent thread in many narratives was the perception of assuming full responsibility for managing the disease in a context with limited external support. Participants frequently referred to gluten-free food as their “treatment” and lamented its high cost: *“Our treatment is food, and it’s much more expensive.”* This was not framed as a minor inconvenience but as a significant economic burden: *“Not everyone can afford it.”* The notion that *“eating without gluten is not a choice — it’s a necessity”* highlighted the medical necessity of the diet and underscored the discordance between individual needs and systemic provision.

Beyond financial strain, several women expressed dissatisfaction with the lack of ongoing professional guidance or institutional recognition, as captured in the simple but resonant plea: *“There should be help.”* Together, economic strain and limited institutional support reinforced the perception that responsibility for disease management rests almost entirely on the individual, amplifying both practical demands and emotional burden.

**Fig. 1:**
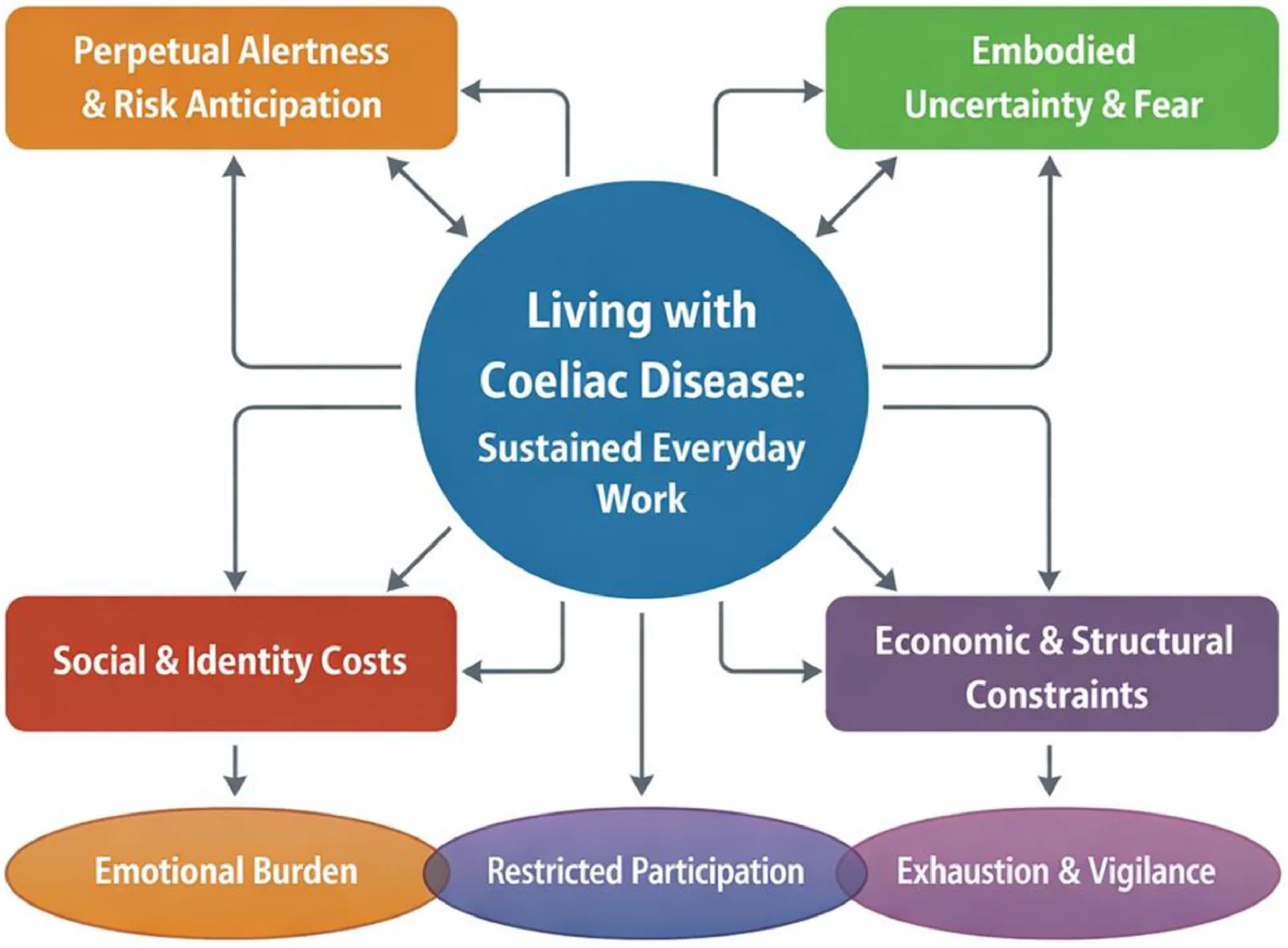
Conceptual model illustrating the interconnected themes identified in the qualitative analysis and their contribution to the experience of living with coeliac disease as sustained everyday work.

Fig. 1 summarizes the four themes emerging from the qualitative analysis and complements Table 1 by visually illustrating how these themes interrelate within participants’ narratives of everyday life with coeliac disease.

## 4 Discusion

We have provided a qualitative interpretation of how women living with coeliac disease in Spain experienced everyday life as sustained, ongoing work rather than as a discrete dietary adjustment. Using free-text narratives from a large survey, we have shown that the burden of the condition was organized around continuous vigilance, embodied uncertainty, social and identity-related costs, and limited structural support. Together, these patterns illustrate how disease management may permeate physical, emotional, and social domains, extending well beyond biomedical notions of dietary adherence.

Across narratives, participants described (i) a clear boundary between “safe” private spaces and “risky” public environments, (ii) persistent vigilance that becomes emotionally exhausting, (iii) social restriction and stigma linked to eating with others, and (iv) structural pressures—particularly financial cost and limited institutional support—that compound day-to-day disease management. These four interconnected themes illuminated how everyday life with coeliac disease was organized around risk, responsibility, and ongoing social negotiation, consistent with qualitative syntheses emphasizing the psychosocial burden of the gluten-free diet beyond symptom control (Möller, Apputhurai, et al., 2021; Kowalczuk and Moor, 2025; Maddison‐Roberts, Jones and Satherley, 2025).

The first theme—feeling safe at home but vulnerable outside—closely aligns with prior qualitative research identifying eating outside the home as a central site of risk appraisal, mistrust, and constrained participation, particularly when individuals cannot verify ingredients or food-handling practices (Peters et al., 2020; Rose, Law and Howard, 2024). This finding also resonates with coeliac disease–specific patient-reported outcome research, in which domains such as worries, concerns, stigma, and social isolation are consistently identified as core components of adult experience, reinforcing the importance of perceived safety in everyday life (Crocker, Jenkinson and Peters, 2018).

The second theme—constant vigilance and emotional exhaustion—echoes qualitative synthesis findings demonstrating that dietary management requires sustained attention, planning, and cognitive effort, often experienced as intrusive and tiring (Rose, Law and Howard, 2024; Kowalczuk and Moor, 2025). We have extended this literature by showing that vigilance operates not only at the level of food choice but also through anticipation of social friction, management of disclosure, and calibration of trust in others. This supports existing evidence that psychosocial processes, rather than dietary adherence per se, are central to the lived burden of coeliac disease (Möller, Hayes, et al., 2021).

Although the concept of “sustained everyday work” was intentionally used to capture the ongoing organisational and psychosocial demands associated with coeliac disease management, participants’ narratives predominantly framed this work as burdensome, emotionally exhausting, and socially constraining rather than neutral or empowering. Some narratives suggested partial adaptation over time, particularly in relation to developing routines or greater familiarity with gluten-free management. However, the broader burden associated with vigilance, social negotiation, and perceived responsibility generally appeared to persist across different stages of living with the condition.

The third theme—social restriction, stigma, and feeling like a burden—fits closely with stigma-oriented qualitative work in coeliac populations, where disclosure dilemmas, minimization by others, and the interpersonal cost of “being difficult” contribute to withdrawal and reduced participation (Olsson et al., 2009; Rose, Law and Howard, 2024). That stigma persists, despite the medical necessity of dietary restriction, reinforces the understanding of coeliac disease as socially negotiated and identity-relevant, rather than merely biomedical. Although much of the stigma literature has focused on adolescents, we suggest that similar mechanisms—concealment, disclosure management, and fear of judgement—continue into adulthood (Olsson et al., 2009; Rose, Law and Howard, 2024).

The fourth theme—economic burden and limited institutional support—reflects accumulating evidence that the cost and accessibility of gluten-free foods, as well as the extent of ongoing healthcare support, shape everyday experience and perceived well-being (Peters et al., 2020; Kowalczuk and Moor, 2025) . In participants’ narratives, the recurrent framing of food as “treatment” was closely intertwined with perceptions of unfairness and abandonment, aligning with research showing variability in follow-up and support, with tangible consequences for patient experience and self-management (Peters et al., 2020; Jeanes et al., 2024).

Although sex and gender may shape chronic illness experience, this qualitative dataset was not generated through purposive sampling or a design intended to examine gendered trajectories; it therefore cannot support robust subgroup comparisons. Accordingly, our analytic focus was on identifying shared patterns of meaning across narratives rather than on between-group contrasts, which is consistent with thematic approaches when data are not collected for comparative analysis (Kiger and Varpio, 2020; Norberg et al., 2023) .

Nevertheless, researchers have suggested possible sex differences in perceived burden and stigma-related experiences associated with coeliac disease management, particularly in relation to emotional burden, social eating, and everyday responsibility for dietary vigilance (Sverker et al., 2009; Rodriguez-Almagro, 2019). Future interview-based research specifically designed to explore these experiences in greater depth would, therefore, be warranted (Hallert et al., 2002).

Free-text survey responses offer a distinct form of qualitative evidence: they capture breadth of experience at scale, allowing identification of recurrent concerns and shared patterns across many respondents, while typically offering less depth than interviews. Recent methodological work supports the legitimacy of analyzing open-ended survey data using thematic approaches, provided that limitations are transparently acknowledged (Cunningham and Wells, 2017; Dalal Albudaiwi, 2017; Abraham et al., 2020; Chintakrindi et al., 2022; Neate et al., 2022; Steele et al., 2025). In the context of coeliac disease, such narratives are particularly valuable for revealing everyday “pressure points” in real-world management that may remain underrepresented in clinic-based qualitative samples (Peters et al., 2020; Rose, Law and Howard, 2024) .

### 4.1 Implications for practice and policy

From these findings, we have identified several actionable implications. First, clinical caregivers should consider recognizing the gluten-free diet as both a dietary and psychosocial intervention target. Ongoing support could address eating-out risk management, disclosure communication, and coping with vigilance-related fatigue, rather than focusing exclusively on dietary compliance (Crocker, Jenkinson and Peters, 2018; Möller, Apputhurai, et al., 2021, 2021) . Second, healthcare services might acknowledge stigma and social restriction as modifiable burdens, through education for families and food-service environments and through follow-up models that legitimize psychosocial challenges, rather than framing them as individual failings (Crocker, Jenkinson and Peters, 2018; Rose, Law and Howard, 2024) . Third, policy attention to affordability and equitable access to gluten-free foods may be warranted not only on nutritional grounds but also because economic pressure amplifies distress and restricts participation—key drivers of reduced well-being identified in qualitative syntheses (Jeanes et al., 2024; Kowalczuk and Moor, 2025).

#### 4.1.1 Strengths and limitations

A key strength of this study is the breadth of narratives captured within a large survey sample, enabling identification of robust, recurring experiential patterns that both align with and extend existing qualitative syntheses (Rose, Law and Howard, 2024; Kowalczuk and Moor, 2025) . The use of an established thematic analytic framework further supports interpretive rigor (Kiger and Varpio, 2020).

Limitations are inherent to free-text data: response length and detail vary, opportunities for follow-up clarification are absent, and the dataset is not suited to fine-grained trajectory analysis or subgroup comparison (Norberg et al., 2023). Because saturation is a debated and method-dependent concept—particularly for non-interview datasets—we interpret analytic adequacy here as the emergence of stable, repeated patterns across a large corpus, rather than claiming classical interview saturation (Malterud, Siersma and Guassora, 2016; Hennink, Kaiser and Marconi, 2017). Future researchers using purposive sampling and in-depth interviews could further examine the mechanisms identified here, including potential gendered and socioeconomic differences (Hallert et al., 2002) . In this sense, coeliac disease emerges not as a static condition to be managed but as an ongoing relational process negotiated across bodies, spaces, and social systems.

## 5 Conclusions

From this qualitative analysis of free-text narratives, we have demonstrated that living with coeliac disease in Spain was commonly experienced by these women as a form of sustained everyday work, characterised by vigilance enacted under social and structural constraints. Experiences of vulnerability outside the home, emotional fatigue arising from constant monitoring, stigma-driven social restriction, and the economic and institutional conditions surrounding access to gluten-free foods intersected rather than operated in isolation, converging to shape everyday life with the condition within this sociocultural context.

Taken together, we have underscored that the burden of coeliac disease extends beyond dietary management and may be deeply embedded in psychosocial and contextual processes. These may call for nursing, health services, and policy responses that move beyond dietary instruction, supporting more holistic models of care that explicitly address emotional labour, social participation, and structural inequities shaping everyday disease management, rather than focusing solely on dietary education or clinical compliance.

## Funding

This study is part of the research project SBPLY/23/180225/000018, funded by the European Union through FEDER and the Regional Government of Castilla-La Mancha (JCCM) through INNOCAM.

## Ethical approval and consent to participate

This study was approved by the **Social Research Ethics Committee (CEIS)** of the **University of Castilla-La Mancha** (Registration No.: **CEIS-2024–36901**) and was conducted in accordance with the principles of the **Declaration of Helsinki** and its subsequent revisions. All participants provided informed consent prior to participation.

## Consent for publication

Not applicable. This study is based on anonymized survey data and does not include any identifiable individual information.

## CRediT authorship contribution statement

**Julián Rodríguez-Almagro:** Writing – review & editing, Writing – original draft, Visualization, Validation, Supervision, Software, Investigation, Data curation, Conceptualization. **Cristina Romero-Blanco:** Writing – review & editing, Writing – original draft, Visualization, Validation, Supervision, Software, Investigation, Data curation, Conceptualization. **Antonio Hernández-Martínez:** Writing – review & editing, Writing – original draft, Visualization, Conceptualization. **María Laura Parra-Fernandez:** Writing – review & editing, Writing – original draft, Visualization, Conceptualization. **Maria Dolores Onieva-Zafra:** Writing – review & editing, Writing – original draft, Visualization, Validation, Supervision, Project administration, Formal analysis, Conceptualization. **Alberto Bermejo-Cantarero:** Writing – review & editing, Writing – original draft, Visualization, Resources, Methodology, Formal analysis, Conceptualization.

## Declaration of competing interest

The authors declare that they have no known competing financial interests or personal relationships that could have appeared to influence the work reported in this paper.

## Data Availability

The datasets generated and/or analyzed during the current study are available from the corresponding author upon reasonable request, subject to ethical and data protection restrictions.
